# Nanocarrier Drug Release and Blood-Brain Barrier Penetration at Post-Stroke Microthrombi Monitored by Real-Time Förster Resonance Energy Transfer

**DOI:** 10.1021/acsnano.4c17011

**Published:** 2025-04-03

**Authors:** Igor Khalin, Nagappanpillai Adarsh, Martina Schifferer, Antonia Wehn, Valeria J. Boide-Trujillo, Uta Mamrak, Joshua Shrouder, Thomas Misgeld, Severin Filser, Andrey S. Klymchenko, Nikolaus Plesnila

**Affiliations:** † Institute for Stroke and Dementia Research (ISD), LMU University Hospital, LMU Munich, Munich 81377, Germany; ‡ Normandie University, UNICAEN, INSERM UMR-S U1237, Physiopathology and Imaging of Neurological Disorders (PhIND), GIP Cyceron, Institute Blood and Brain @ Caen-Normandie (BB@C), Caen 14000, France; § Laboratory de Biophotonique et Pharmacologie, University of Strasbourg, Strasbourg 60024, France; ∥ Department of Polymer Chemistry, Government College Attingal, Thiruvananthapuram 695101, Kerala, India; ⊥ German Center for Neurodegenerative Diseases (DZNE), Munich 81377, Germany; # Munich Cluster of Systems Neurology (SyNergy), Munich 81377, Germany; ∇ Department of Neurosurgery, LMU University Hospital, Munich 81377, Germany; ○ Institute of Neuronal Cell Biology, Technical University of Munich, Munich 80802, Germany; ◆ Core Research Facilities and Services-Light Microscope Facility, German Center for Neurodegenerative Diseases (DZNE), Bonn 53127, Germany

**Keywords:** blood-brain barrier, stroke, microthrombosis, nanocarriers, correlative light-electron microscopy

## Abstract

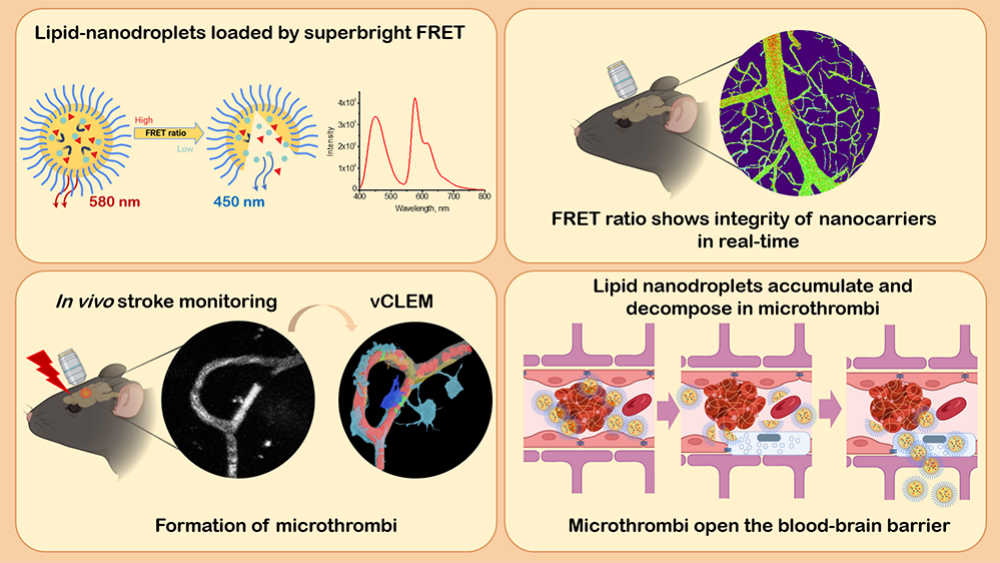

Nanotechnology holds great promise for improving the delivery of therapeutics to the brain. However, current approaches often operate at the organ or tissue level and are limited by the lack of tools to dynamically monitor cargo delivery in vivo. We have developed highly fluorescent lipid nanodroplets (LNDs) that enable tracking of nanocarrier behavior at the subcellular level while also carrying a Förster resonance energy transfer (FRET)-based drug delivery detection system (FedEcs) capable of monitoring cargo release in vivo. Using two-photon microscopy, we demonstrate that circulating LNDs in naïve mouse brain vasculature exhibit 3D real-time FRET changes, showing size-dependent stability over 2 h in blood circulation. Further, in the Nanostroke model, dynamic intravital two-photon imaging revealed that LNDs accumulated within cerebral postischemic microthrombi, where they released their cargo significantly faster than in normal blood circulation. Furthermore, the blood-brain barrier (BBB) became permeable at the microclot sites thereby allowing accumulated FedEcs-LNDs to cross the BBB and deliver their cargo to the brain parenchyma. This microthrombi-associated translocation was confirmed at the ultrastructural level via volume-correlative light-electron microscopy. Consequently, FedEcs represents an advanced tool to quantitatively study the biodistribution and cargo release of nanocarriers at high resolution in real-time. By enabling us to resolve passive targeting mechanisms poststroke, specifically, accumulation, degradation, and extravasation via poststroke microthrombi, this system could significantly enhance the translational validation of nanocarriers for future treatments of brain diseases.

## Introduction

Brain diseases, including stroke, brain trauma, neurodegenerative disorders, and brain tumors, pose significant global health challenges due to their high morbidity and mortality rates.^1^ Effective treatment of these conditions is often hindered by the blood-brain barrier (BBB), a highly selective barrier that restricts the entry of most therapeutics into the brain parenchyma.^2^ Overcoming the BBB to deliver drugs effectively remains a critical hurdle in neuroscience and pharmacology.

Nanotechnology is currently revolutionizing the delivery of therapeutics by offering innovative solutions to transport drugs to target organs. Along with widely used lipid nanoparticles (LNPs), lipid nanodroplets (LNDs), also known as nanoemulsions, are particularly attractive and promising platforms because of their biocompatibility, structural similarity to low-density lipoproteins, and favorable pharmacokinetic properties.^3^ The liquid core serves as an excellent reservoir for encapsulation of lipophilic drugs and contrast agents,^3^ while their lipid shell is biocompatible and therefore generally recognized as safe (GRAS) for application in patients.^4−6^ LNDs are characterized by prolonged circulation time in blood, which enhance their potential for both passive and active tissue targeting.^7^ Previous studies have demonstrated that LNDs can remain intact in blood circulation for several hours postinjection and preferentially accumulate in tumors due to the enhanced permeability and retention (EPR) effect. However, despite these advantageous properties, investigating the biodistribution of LNDs and other nanoparticles (NPs) in the brain remains a fundamental challenge. Current detection modalities lack the resolution and specificity to dynamically monitor whether NPs cross the BBB, enter the brain parenchyma, and release their therapeutic cargo at the specific site.^2^ To overcome this limitation, we developed highly fluorescent LNDs capable of being detected in vivo with a high spatial resolution. Moreover, our LNDs carry a Förster-resonance energy transfer (FRET)-based drug delivery detection system (FedEcs) which allows for real-time visualization of cargo release in vivo. Additionally, the lipid core of LNDs made them visible in electron microscopy (EM), enabling us to establish a powerful correlative light-electron microscopy (CLEM) method. This method allows us to combine dynamic changes observed intravitally with ultrastructure insights into the BBB leakage.

Using advanced imaging techniques such as in vivo 2-photon microscopy and volume CLEM, we investigated the biodistribution and cargo release of FedEcs-LNDs in a mouse model of cerebral ischemia induced by magnetic nanoparticles (Nanostroke model). Our observations revealed that 30 nm FedEcs-LNDs accumulate within microthrombi forming in brain capillaries poststroke, cross the compromised BBB at these sites, and release their cargo both within the microthrombi and brain parenchyma. These findings demonstrate the potential of LNDs for passive targeting of the ischemic brain, crossing the BBB at microthrombi, and highlight the importance of subcellular-level real-time monitoring for the development of effective nanomedicines.

## Results

### Development of Highly Fluorescent Lipid Nanodroplets (LNDs) Carrying a Förster-Resonance Energy Transfer (FRET)-Based Cargo Delivery Detection System (FedEcs)

**Figure 1:**
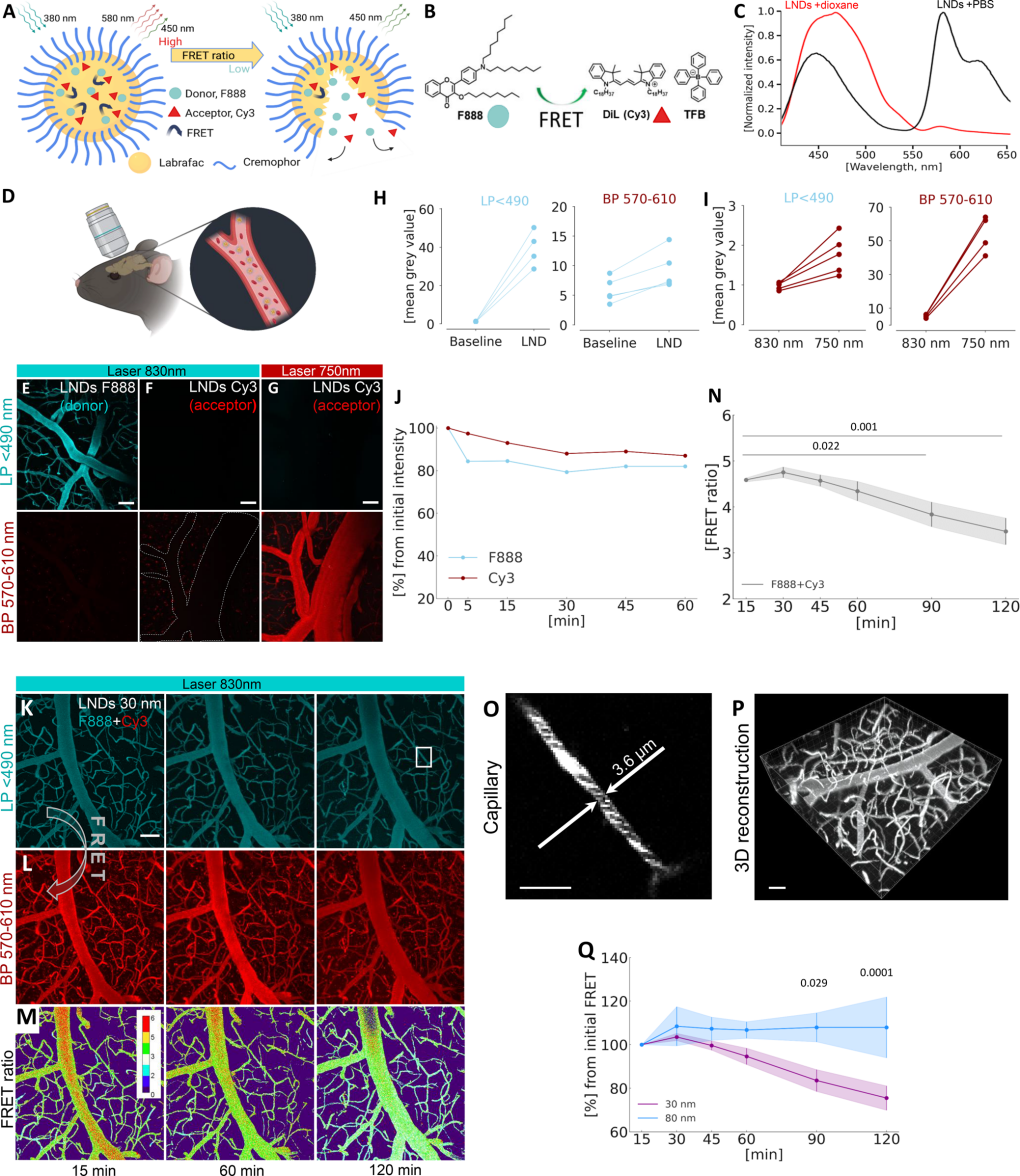
Design, characterization, and in vivo evaluation of lipid nanodroplets (LNDs) for real-time monitoring of drug release. (A) Schematic representation of Förster-resonance energy transfer (FRET) nanocarrier that can shift its color upon decomposition, thus reducing the total FRET ratio. The LND consists of an oil core (yellow; Labrafac WL), surfactant (blue; Cremaphore, Kolliphor ELP), and the fluorescent FRET pair dyes F888 (blue sphere) and Cy3 (red triangle) bound to a hydrophobic counterion. (B) Chemical structures of F888 and Dil (Cy3) coupled with a counterion tetraphenylborate, responsible for causing the FRET effect. (C) In vitro fluorescence spectrum of FRET-LNDs containing F888 and DiI-TPB (2 wt % loading each) diluted in PBS (back) and dioxane (red). (D) Schematic drawing of intravital imaging of mouse cortical vessels performed via the cranial window in the skull. (E–G) Representative in vivo 2-photon images of mouse cortical vessels after injection of 30 nm LNDs loaded solely by F888 (donor; excitation laser wavelength 830 nm; (E) or Cy3 (acceptor; excitation laser wavelength 830 and 750 nm; (F,G). Maximum intensity projection (MIP) of 150 μm Z-stacks; laser power 4.5–13%; donor channel–LP < 490 nm; acceptor channel–BP 570–610 nm; scale bar −50 μm. (E) Bright fluorescent signal was observed in the donor channel at 830 nm. (F) Absence of a signal in both the donor and acceptor channel. Dashed line outlines an invisible vessel, which becomes visible only at 750 nm. (G) Bright fluorescent signal was observed in acceptor channel. (H) Quantification of increased fluorescence intensity after injection of donor-loaded LNDs (*n* = 5 regions of interest in the vessels) in the donor (LP < 490) and acceptor (BP 570–610 nm) channel. (I) Fluorescence intensity increase observed in acceptor-loaded LNDs after shifting the laser wavelength from 830 to 750 nm in both the donor and acceptor channels (*n* = 5 regions of interest in the vessels). (J) Elimination rates of LNDs loaded by F888 (donor) and Cy3 (acceptor) from the blood circulation of naïve mice during 1 h (*n* = 1). (K,L) Representative real-time in vivo two-photon MIP images of mouse cortical vessels captured during 15–120 min after injection of 30 nm FedEcs-LNDs loaded by FRET pair (F888+Cy3), excitation laser wavelength 830 nm. The acceptor became visible via BP 570–610 detector due to the FRET effect at an excitation laser wavelength of 830 nm. Scale bar −50 μm. (M) Color code of FRET ratiometric changes in circulated FRET-LNDs. Scale bar −50 μm. (N) Quantification of time-dependent LNDs disintegration in the mouse brain vasculature measured by changes in FRET ratio. Data are presented as mean ± standard deviation (SD). One-way ANOVA was used, *n* = 3. (O) High resolution image showing distribution of LNDs within capillaries and between circulated blood cells. Scale bar: 20 μm. P. 3D reconstruction of real-time imaging of circulated LNDs in brain vasculature. Scale bar −50 μm. (Q) Comparative analysis of the degradation of circulated LNDs of two different sizes (30- and 80 nm) using FRET ratio. Data presented as mean ± SD. Two-way ANOVA was used, *n* = 3. Created with BioRender.com.

We aimed to design highly fluorescent nanodroplets that employ FRET to dynamically assess both the structural integrity of the particles and the release of their encapsulated cargo. To achieve this, LNDs were formulated using LabrafacWL and Cremophor ELP through spontaneous nanoemulsification.^9^ These LNDs were loaded with two fluorophores capable of FRET and detecting the LNDs decomposition: upon degradation of the LNDs and/or cargo release, the distance between the donor and acceptor dyes increases, thereby leading to the loss of FRET and a subsequent reduction of the ratio between donor and acceptor fluorescence intensity (Figure 1A). The fluorescent dye F888, which was specially designed for high loading into LNDs and for superior fluorescence intensity following 2-photon excitation,^10^ was selected as the FRET donor and the Cy3 derivative DiI was selected as the FRET acceptor. To ensure efficient encapsulation inside LNDs with minimal dye leakage, DiI was coupled to tetraphenylborate (TPB)^11^ (Figure 1B; Suppl. Figure S1A). The LNDs thus function as FRET-based cargo delivery detection systems (FedEcs). In contrast to previously reported FRET-LNDs operating in the NIR region for whole mice imaging, here we propose FRET-LNDs specially designed for 2-photon intravital imaging. This configuration enables targeted three-dimensional (3D) imaging of nanoparticles circulating within the brain vasculature at micrometer spatial resolution and subsecond temporal resolution. LNDs with a diameter of 30- and 80 nm, as measured by dynamic light scattering, containing either the donor, acceptor, or both dyes were formulated (Suppl. Figure S1B). At an excitation wavelength of 380 nm, LNDs of both sizes showed the fluorescent properties of the donor, i.e. fluorescence emission at 450 nm, as well as a second fluorescence peak at 580 nm, corresponding to the expected emission of the acceptor due to FRET (Suppl. Figure S1C,D). The composition of the generated LNDs consisted of 98% lipids with the remaining 2% accounted for by the donor and acceptor fluorophores. At this weight, FRET efficiency between the donor and acceptor reached 86% for 30 and 80 nm LNDs (Suppl. Figure S1E), indicating a highly effective energy transfer between the fluorophores. Dilution of FedEcs-LNDs into dioxane, a solvent known to disrupt the LND structure, led to the loss of FRET, as indicated by a fluorescence emission dominated by the donor dye alone (Figure 1C). These data demonstrate that our FRET system can reliably detect LNDs integrity and monitor cargo delivery.

### Using FedEcs To Monitor the Stability of Circulating LNDs in Healthy Brain Vessels In Vivo

To investigate the critical question of whether FedEcs-LNDs are bright enough to be visualized in vivo and to validate their functionality, we injected FedEcs-LNDs systemically into mice and investigated the cerebral vasculature by in vivo 2-photon microscopy (2-PM, Figure 1D). The FedEcs-LNDs exhibited strong fluorescence, allowing for clear visualization in vivo. As expected, LNDs loaded with either the donor (F888) or acceptor (Cy3-TPB) fluorophores emitted fluorescence only when excited at specific wavelengths for each fluorophore (830 nm for F888 and 750 nm for Cy3-TPB; Figure 1E–I). Longitudinal imaging confirmed the stability of the circulated LNDs in vivo loaded with either fluorophore individually (Figure 1J). LNDs loaded with both FRET donors and acceptors (FedEcs-LNDs) emitted bright fluorescence at 830 nm in both channels (Figure 1K,L), corroborating the in vitro data and confirming that FRET occurs in vivo. This finding suggests that FRET can be dynamically assessed in real-time, allowing us to monitor particle integrity by quantifying the FRET ratio, expressed as the acceptor-to-donor fluorescence intensity ratio (Figure 1M). Real-time analysis of the FRET ratio of circulating 30 nm LNDs in healthy mice revealed a slow decrease in FRET, which dropped from 4.6 to 3.5 of the FRET ratio value over 120 min (Figure 1N). Notably, the spatial resolution of monitored FedEcs-LNDs was enough to observe the motion of nanoparticles within the capillary and between blood cells and across the 3D volume of the brain (Figure 1O,P). Interestingly, 80 nm LNDs had a substantially higher (*p* = 0.0001) stability in the blood circulation, remaining intact for two h, while 20% of 30 nm LNDs degraded within the same time frame (Figure 1Q). This enhanced stability is likely due to the higher oil-to-surfactant ratio in the larger particles. These findings demonstrate that our system can dynamically assess the real-time integrity of circulating nanoparticles using the FRET ratio. To our knowledge, this is the first report of a nanoparticle system able to produce a stable FRET signal in vivo, suitable for longitudinal high-resolution intravital 2-PM.

### Employing FedEcs To Define the Distribution of LNDs after Ischemic Stroke In Vivo

**Figure 2:**
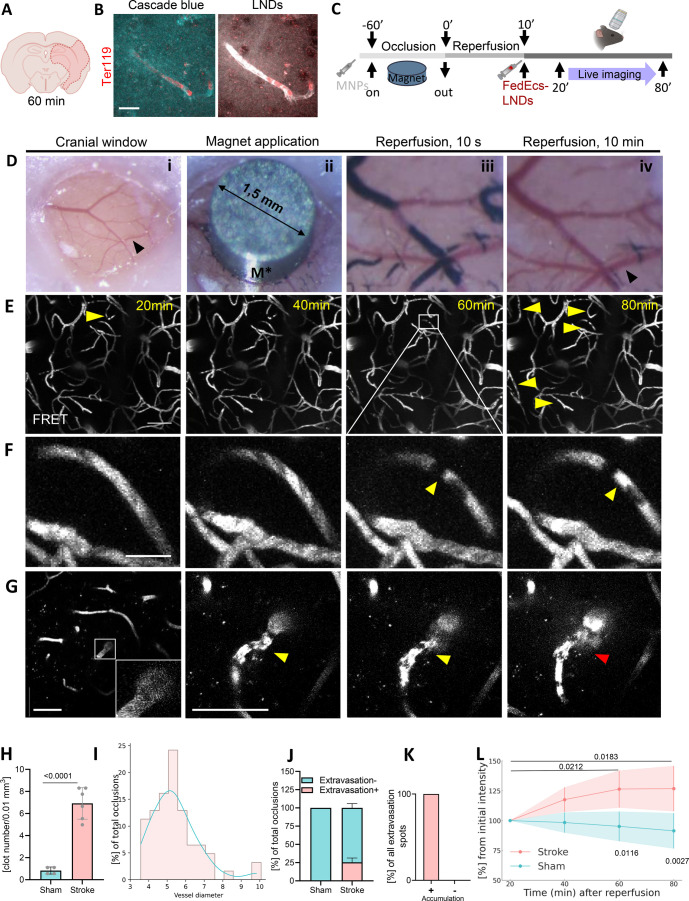
Spontaneous poststroke microclot formation opens the blood-brain barrier for LNDs. (A) A filament middle artery occlusion (fMCAo) during 60 min followed by injection of 30 nm LNDs loaded with rhodamine were performed as described at (Suppl. Figure S2A,B). (B) Representative confocal image of the stroke-affected area in a mouse brain showing extravasation in the microclot region. Stalling erythrocytes in the microclot area are stained with the Ter119 antibody (red), allowing a comparison of the extravasation of Cascade Blue 3000 Da dextran versus LNDs. Scale bar −20 μm. (C) Nanostroke model experimental design. A mini magnet was placed for 60 min on the top of the glass-covered cranial window. Magnetic nanoparticles (MNPs) were injected systemically causing the occlusion of the vessels underneath the magnet. Twenty min after removal of the magnet, the animal was placed under the two-photon microscope (2-PM) for live imaging. (D) Macrophotographs of different stages of the stroke model. (i) Glass-covered cranial window; black arrow–middle cerebral artery (MCA). (ii) Micromagnet (M*; diameter 1.5 mm) placed on top of the cranial window located above the territory of the MCA. (iii) MNPs accumulated in the vessel lumen of cerebral vessels underneath the magnet (black). iv vessels reperfused within 10 min after removal of the magnet; black arrow–MCA. (E) Intravital 2-PM images of cortical vessels of the mouse with Nanostroke model injected with FedEcs-LNDs. Yellow arrows–spontaneous vascular occlusions. Scale bar −50 μm. (F) Zoomed from (E), real-time images of a representative spontaneous microvascular clot formation with subsequently accumulated LNDs (yellow arrow). Scale bar −10 μm. (G) Representative longitudinal real-time images of spontaneous microvascular clot formation followed by extravasation of LNDs into the brain parenchyma (white arrow–flowing microvessel; yellow arrow–occluded; red–with extravasation). Scale bar −50 μm. (H) Analysis of numbers of microvascular clots at 80 min postreperfusion compared to sham animals. Data are presented as mean ± standard deviation (SD). Unpaired *t* test. Stroke: *N* = 79 clots (6 mice); Sham −4 mice. (I) Size distribution of the vessels with microclots. Data are presented as proportion of vessel diameter where microclots were detected. *N* = 62 clots (6 mice). (J) Analysis of the Association between microclot (accumulation) and blood-brain barrier dysfunction (extravasation). *N* = 20. (K) Proportion of microvascular clots associated with extravasation of the LNDs. Data are represented as mean ± SD *N* = 4 (sham) and *n* = 6 (Nanostroke). (L) Longitudinal analysis of the accumulation of FedEcs LNDs in the ischemic brain area. Data represented as mean ± SD. Two-way ANOVA was used, *n* = 4 (sham) and *n* = 6 (Nanostroke). Created with BioRender.com.

To investigate the behavior of LNDs in the brain under pathological conditions, we induced focal cerebral ischemia by occluding the middle cerebral artery of a mouse with an endovascular filament for one h and systemically injected highly fluorescent LNDs 30 min after reperfusion (Figure 2A; Suppl. Figure S2A,B). We focused on 30 nm LNDs, due to their prolonged blood circulation and optimal size, which enables them to cross the BBB via transcytosis, as shown in our recent study.^12^ Brain tissue was harvested 60 min after LND injection, and we detected widespread accumulations of LNDs within the ischemic territory (Suppl. Figure S2C). Staining of blood vessels (lectin) and red blood cells (Ter119) demonstrated that LNDs were located within cerebral capillaries and colocalized with stalled erythrocytes. Interestingly, while the conventional fluorescent tracer Cascade Blue (3000 Da), coinjected with LNDs, was diffusively distributed in the lesion area, the distribution of LNDs had a specific pattern: the intensity peak inside of microclots and a high-intensity area in the proximity of the vessel occlusions (Figure 2B; Suppl. Figure S2C, i–iii). Importantly, no LND fluorescent signal was detected in the contralateral hemisphere of the brain, suggesting that LNDs do not exhibit spontaneous accumulation or cross an intact BBB. This implies that 30 nm LNDs may passively accumulate in the ischemic region inside microthrombi and extravasate into the brain parenchyma.

To further investigate this phenomenon, we then aimed to analyze LND biodistribution in real-time and with the highest possible resolution to decipher the route/mechanism by which LNDs extravasated and whether the accumulation of LNDs in the ischemic brain may have affected their ability to carry and deliver cargo. For this purpose, we developed a targeted stroke model based on a technique previously used for inducing focal cerebral ischemia in juvenile mice.^13^ This model employs iron oxide magnetic nanoparticles (MNP) to transiently occlude cortical blood vessels. Following occlusion, the animal was placed under 2-PM for in vivo imaging of the affected cortical area (Figure 2C). In detail, animals received a systemic injection of MNP and subsequently, a strong mini-magnet was placed on top of the implanted cranial window to occlude vessels of the MCA territory (Figure 2D,i). The magnetic field trapped the 180 nm pegylated MNPs within the cerebral vasculature (Figure 2D,ii) thereby causing cerebral ischemia (Nanostroke). After 1 h, removing the magnet allowed the MNPs to wash away, mimicking recanalization after stroke and allowing tissue reperfusion (Figure 2D,iii–iv). Using EM, we were able to resolve single iron oxide particles and demonstrate that, during occlusion, MNPs caused localized astrocytic swelling (Suppl. Figure S3A) without damaging the vessel intima (Suppl. Figure S3B), indicating ischemic tissue damage. This model occluded cortical blood vessels up to a depth of 500 μm (Suppl. Figure S3C) and allowed immediate live 2-PM of reperfused cerebral vessels (Suppl. Figure S3D). Analysis of microglia stained with Iba-1 (Figure S3E,F) in the region beneath the magnet revealed increased overall coverage and a higher circularity index in the ipsilesional hemisphere compared to the corresponding area in the contralesional hemisphere. Additionally, reduced cell ramification was observed, which was collectively indicative of microglial activation. Furthermore, the number of neurons (Suppl. Figure S3G,H), identified by NeuN staining, was significantly reduced in this region, while astrocytes exhibited extensive GFAP expression (Suppl. Figure S3I). Altogether, these findings demonstrate that the area under the magnet exhibited an inflammatory response and neuronal loss, consistent with lesion formation in the brain cortex. Using the Nanostroke model and FedEcs-LNDs, we investigated the passive biodistribution of systemically applied LNDs in the brain after experimental stroke by in vivo 2-PM, focusing first on LNDs with preserved integrity according to their FRET (acceptor) signal (Figure 2E). Postreperfusion, we observed LND accumulations within nonflowing segments of cerebral vessels increasing during imaging (Figure 2E, yellow arrows). These accumulations were spatially associated with nonmoving black cell shadows, which we consider as microvascular occlusions or microthrombi. It is important to note that some LND accumulations were newly formed or spontaneous (Figure 2F,G, yellow arrow), while some were also accompanied by subsequent extravasation of LNDs into the brain parenchyma (Figure 2G, 80 min, red arrow). Quantification showed that the number of LND accumulations 80 min after ischemic stroke was 8-fold higher than in sham-operated mice (Figure 2H; *p* < 0.0001; Suppl. Figure S4) and that 90% of all observed occlusions occurred in vessels less than 6 μm, *i.e*. brain capillaries (Figure 2I), further implying that LNDs were trapped in microvascular-occlusions. Interestingly, 25% of all microthrombi observed after stroke were associated with extravasation of LNDs (Figure 2J), while no extravasations were detected in sham-operated animals. Most importantly, extravasation of LNDs was only observed at sites where micro-occlusions formed (Figure 2K), suggesting that micro-occlusions compromised the permeability of the adjacent capillary wall and were critically involved in the opening of the BBB. Longitudinal in vivo imaging of LNDs allowed us to detect particles that gradually accumulated within the ischemic brain (Figure 2L). Altogether, following ischemic stroke, 30 nm LNDs passively accumulated within the vessel lumen around the microclot over 80 min and also extravasated from there into the ischemic brain parenchyma. Our data provide the first visualization of LNDs entering the ischemic brain at microthrombi sites, revealing a new avenue to deliver drugs into ischemic tissue.

**Figure 3:**
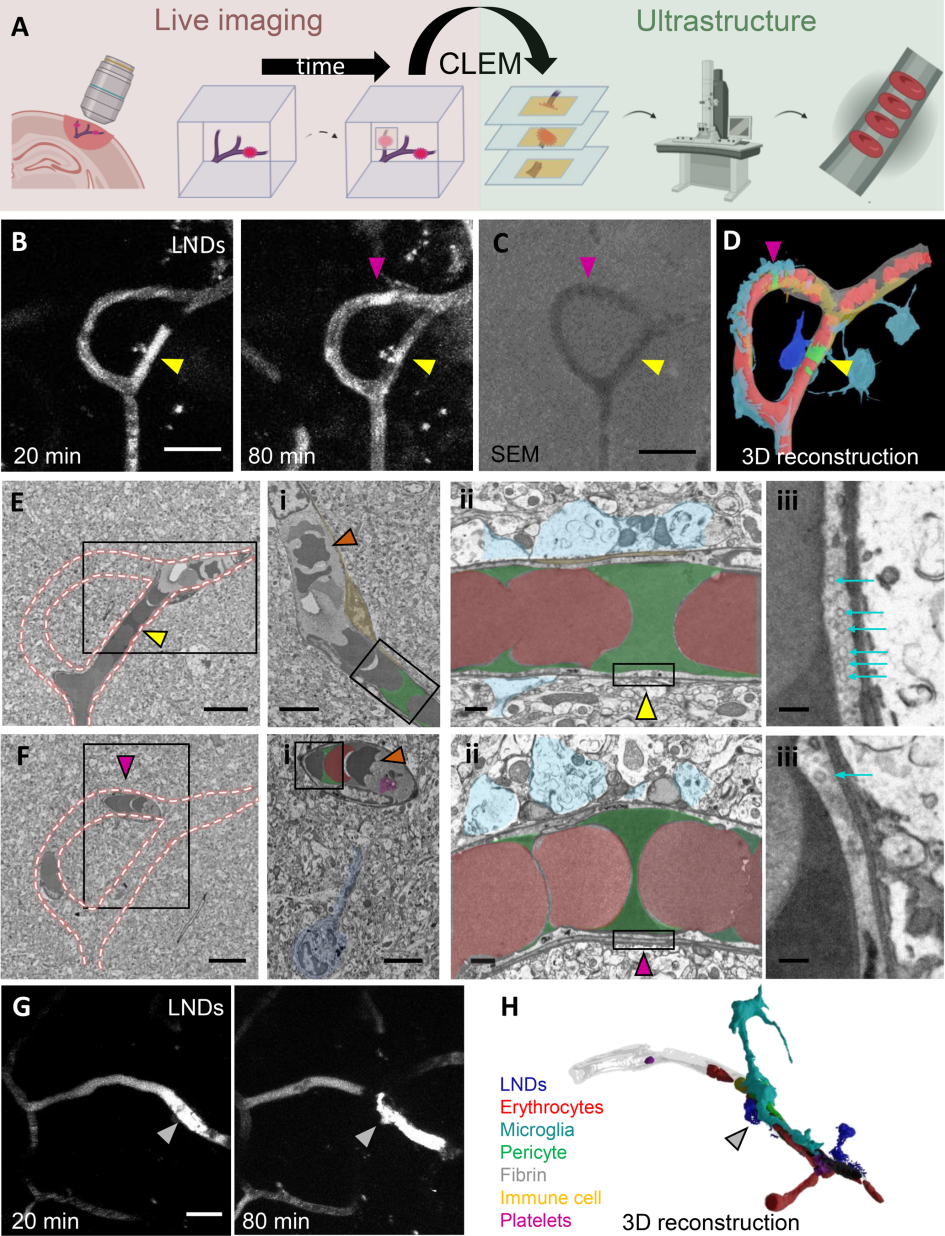
Correlative light-electron microscopy revealed ultrastructural changes in sites of accumulation and extravasation of FedEcs-LNDs. (A) Experiment design as described in Suppl. Figure S3A with longitudinal intravital imaging during 60 min followed by fixation, embedding and serial sectioning for correlated volume electron microscopy (EM) by automated serial sections to tape (ATUM; Suppl. Figure S5). (B) Representative 2-PM images of poststroke mouse brain capillaries containing “old” (appeared at 20 min postreperfusion; yellow arrow) and “fresh” (80 min; magenta arrow) spontaneous microvascular clots. Scale bar 20 μm. (C) Summed slices of a scanning electron microscopy (SEM) stack of the relocated region from the 2-PM experiment (Suppl. Figure S5). Scale bar 20 μm. (D) Rendered three-dimensional reconstruction of the volume SEM data thereof. Red (erythrocytes); yellow (pericytes); green (LNDs); sky blue (astrocytes); dark blue (microglia); magenta (platelet). (E,F) Single segmented high-resolution SEM images of the “old” (E; yellow arrow) and “fresh” (F; magenta arrow) microclots relocated from 3B. Scale bar 10 μm. (E,i) Zoomed area from (E) containing an “old” microclot with accumulated LNDs (green) and a pericyte constricting the capillary (yellow). Orange arrow–dysmorphic erythrocytes trapped into fibrine. (E,i). Zoomed area from (E,i) containing an occluded capillary with accumulated LNDs (green) between erythrocytes (red). Sky blue–astrocytic end feet. Yellow arrow–relocated capillary area with accumulated LNDs showing a higher than in flowing capillaries fluorescent intensity in 2-PM. Scale bar −1 μm. (E,iii) Zoomed brain endothelium cell (BEC) from (E,ii) containing a large number of vesicular-like structures (caveolae; cyan arrows). Scale bar −200 nm. (F,i) Zoomed area from (F) containing a “fresh” microclot with accumulated LNDs (green) between erythrocytes (red) and microglia cell (dark blue) with its processes directed toward the platelet cells (magenta) trapped into fibrine. (F,ii) Zoomed area from (F,i) containing an occluded capillary with accumulated LNDs (green) between erythrocytes (red). Sky blue–astrocyte end feet. Magenta arrow–relocated capillary area with accumulated LNDs having higher fluorescent intensity in 2-PM. Scale bar −1 μm. (F,iii) Zoomed BEC from (F,ii) with lower level of caveolae-like structures (cyan arrow). Scale bar −200 nm. (G) Representative example of a microvascular clot (20 min; gray arrow) transitioning toward LNDs extravasation (80 min) into the brain parenchyma (“leaky” microclot). Scale bar 20 μm. (H) Rendered three-dimensional reconstruction of volume electron microscopy of the relocated region from Figure 3G. Red–erythrocytes; dark blue–LNDs; cyan–microglia; gray–fibrin; green–pericyte; magenta–platelets; yellow–leukocytes; transparent gray–BEC. See in Movie S03. Created with BioRender.com.

To better understand the local distribution of LNDs inside occluded vessels, as well as the morphology and ultrastructure of vascular occlusions and extravasation sites, we developed a unique correlative light-electron microscopy (CLEM) method. Since MNPs are both visible with light microscopy and electron-dense, they can be used as fiducial markers for CLEM. Capitalizing on this feature, we tracked fluorescent emission of LND accumulations and extravasations in real-time using 2-PM in vivo. We then correlated these dynamic observations with ultrastructural data by relocating the fluorescent signal to EM and performing 3D array tomography via automated tape-collecting ultramicrotomy (ATUM) to generate ultrastructural 3D images of the chosen clot (Figure 3A; Suppl. Figure S5; Methods). On 2-PM time-serial Z-stacks of a mouse brain after 60 min stroke, we located two representative microvascular occlusions marked by LND-FedEcs accumulation that appeared at different time points—20 and 80 min after reperfusion (Figure 3B, yellow and magenta arrows). Then, the same occlusions were relocated using CLEM and successfully reimaged with EM (Figure 3C; Movie S01). After full reconstruction of the vessel segments in 3D (Figure 3D; Movie S02), we could pinpoint the cellular composition and microstructure of each occluded vessel at the LNDs accumulation site (Figure 3E,F; yellow arrow–clot appeared at 20 min; magenta −80 min). Microglia and constricted pericytes surrounded the occluded vessels, while erythrocytes, platelets, and LNDs colocalized within the occlusions (Figure 3E,F). When zooming on the ultrastructure of the microvascular occlusions, we found fibrin nets (orange arrow) between red blood cells, further suggesting that the observed occlusions were clearly identified as microthrombi (Figure 3E,F, i). Interestingly, the microclot that formed early after reperfusion according to in vivo imaging contained erythrocytes with polyhedral morphology, while those formed later had erythrocytes with a normal biconcave shape (Figure 3E,F, ii, red). Collectively, these findings show that stroke induces the formation of microthrombi within the cerebral microcirculation and that LNDs became trapped (Figure 3E,F, i, ii, green) during the formation of these microclots.

Further, using CLEM, we precisely investigated the microstructure of brain endothelial cells (BECs) in the proximity of microclots with accumulated LNDs (Figure 3E,F, ii-iii). The BECs in the vicinity of an older clot had a much higher number of caveolae (23 caveolae/μm; Figure 3E, iii; cyan arrows) than BECs next to a fresh microclot (2.8 caveolae/μm; Figure 3F, iii), implying a persisting contact between BECs and clots may promote the formation of caveolae, the prerequisite for the subsequent compromising of the BBB and, as a result, extravasation of LNDs into the brain parenchyma. Immunohistochemical staining confirmed a higher expression of Cav-1 in the proximity of occluded vessels (Figure S6A,B). Additionally, a representative time-lapse series recorded with two-photon microscopy revealed a microclot that became “leaky” 60 min after initial occlusion (Figure 3G, detected by LND accumulation shown gray arrow), providing longitudinal evidence that the persistence of microclots compromises the BBB in their proximity. To confirm this, we performed CLEM on the representative “leaky” clot with accumulated LNDs and extravasation next to it. A 3D reconstruction of the ultrastructure revealed that the extravasation site (Figure 3H, gray arrow) was fully colocalized with microthrombi composed of erythrocytes, platelets, and immune cells (Movie S03). Outside the vessel, the microclot was surrounded by pericytes and microglial processes, resembling the inner and outer compositions of two other aforementioned reconstructed clots. Thus, our electron microscopy data demonstrate that stroke-induced persistent microclots within the cerebral microcirculation progressively create an environment conducive to increased endothelial transcytosis via caveolae, which may be associated with BBB disruption and the subsequent entry of LNDs into the brain parenchyma.

To investigate this process on the functional level in vivo, mice received a systemic injection of LNDs first and were then subjected to the occlusion of the vessels via magnet-MNP interaction for 60 min. The cerebral microcirculation was imaged by in vivo 2-PM before and after occlusion (Suppl. Figure S7). Images obtained immediately after recanalization showed multiple spots of LND extravasation at the occluded area (Suppl. Figure S7, yellow arrows), supporting our histological and EM findings that micro-occlusion of the vessels (even caused by iron oxide nanoparticles) may serve as an entry point for LNDs into the brain parenchyma. Thus, by occluding vessels with MNPs, we were able to locally open the BBB.

### Monitoring Cargo Delivery of LNDs in the Brain Using FedEcs

**Figure 4:**
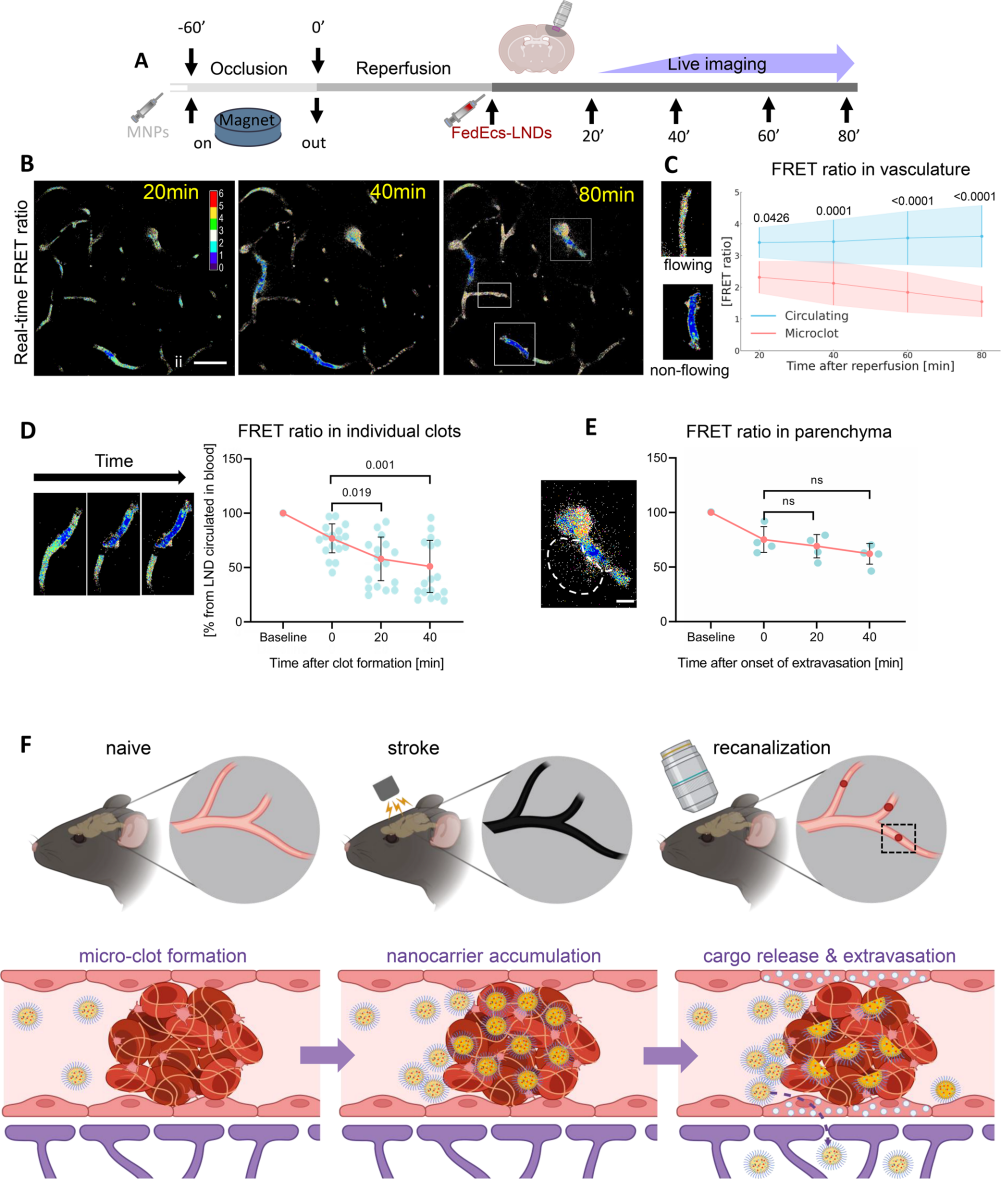
FRET ratio reveals higher decomposition of FedEcs-LNDs in microclots, but not in brain parenchyma as well as delivery of cargo into the brain parenchyma. (A) Experiment design. (B) Representative longitudinal real-time images of visualization of the FRET ratio related to decomposition of FedEcs-LNDs in vasculature of the ischemic brain area. Scale bar −50 μm. (C) Longitudinal analysis FRET ratio related to decomposition of FedEcs-LNDs in vasculature of ischemic brain area during 80 min after recanalization. Data are presented as mean ± SD. Two-way ANOVA was used, circulated *n* = 16–17 ROIs; microclots *n* = 6–13; 5 mice. (D) Time-dependent analysis of relative FRET changes of LNDs located inside individual clot. (E) Time-dependent analysis of relative FRET changes extravasated into brain parenchyma LNDs. Data are presented as single points and mean ± SD. One-way ANOVA was used, *n* = 17 clots and *n* = 4 extravasations, respectively. Scale bar–10 μm. Yellow arrow–clot; dashed area–extravasated FedEcs-LNDs emitting FRET. (F) Schema of the mechanism of nanoparticles crossing the blood-brain barrier via microthrombi. Created with BioRender.com.

The entry of LNDs into the brain parenchyma is, however, not equivalent to cargo delivery. Therefore, we used the newly developed FedEcs functionality of our highly fluorescent LNDs to monitor whether LNDs decompose after being trapped in microclots and transmigrate into the brain parenchyma. To assess this, we performed a Nanostoke (Figure 4A) and measured the FRET ratio in flowing versus nonflowing segments of the microvasculature after cerebral ischemia over time by quantitative in vivo FRET imaging (Figure 4B). While the FRET ratio of LNDs circulating in plasma was stable during 80 min of observation, the FRET ratio of LNDs trapped within microclots was significantly lower and gradually decreased (Figure 4C). The loss of FRET is caused by a loss of interaction between the two fluorophores undergoing FRET and is therefore a quantitative measure of particle decomposition. Hence, our data indicate that LNDs are stable in plasma, but start decomposing once being trapped in microclots. This observation is further confirmed by the analysis of newly formed individual microclots over time, (Figure 4D). Once trapped in a formed microclot, the FRET ratio of LNDs starts declining and reaches about 50% of its baseline value after 40 min. Interestingly, a very similar trend to the decay of FRET ratios can also be observed when focusing the analysis on LNDs locally extravasated into the brain parenchyma (Figure 4E). The presence of a FRET signal in the parenchyma indicates that intact LNDs entered the brain at the site of the microthrombi. Additionally, these LNDs show a constant decay of their FRET ratio, suggesting that LNDs release their cargo, in our case the fluorophores undergoing FRET, after entering the brain parenchyma. A similar decomposition rate inside poststroke microthrombi was detected while using 80 nm FedEcs, however, we did not observe extravasation at the site of the microthrombi (Figure S8A–D).

In summary, using highly fluorescent LNDs and FedEcs, an advanced in vivo real-time cargo release detection system, we unraveled the passive biodistribution of LNDs after ischemic stroke in vivo and at the ultrastructural level (Figure 4F). Our data demonstrated that after a transient ischemic stroke caused by MNPs-magnet interaction, the newly formed microclots are developed in the stroke area. FedEcs lipid nanodroplets accumulated inside this microthrombi, releasing their cargo there, crossing the BBB, and entering the brain parenchyma. Thus, FedEcs represent a valuable tool to investigate the therapeutic potential of nanoparticle-based drug delivery systems and directly visualize cargo delivery in vivo with high spatial and temporal resolution.

## Discussion

Targeted drug delivery using nanotechnology has two conflicting aims: nanoparticles must reliably enclose their cargo and transport it to the target tissue, but release it there effectively.^14^ Both aims are hard to achieve and even more challenging to investigate experimentally due to insufficient technologies for particle tracking and cargo release in living organisms.^2^ In the current study, we developed highly fluorescent LNDs with a built-in cargo release function based on FRET and used these nanoparticles (NPs) to investigate the biodistribution of LNDs and cargo release in a targeted, magnetic NPs-based model of ischemic stroke in mice. One of the key observations in our study was the passive accumulation of 30 nm LNDs within newly formed microclots following transient ischemic stroke. This size of LNDs was specifically chosen due to their demonstrated ability to cross the BBB via transcytosis, as shown in our previous work.^12^ Importantly, our data showed that LNDs not only accumulated within the ischemic territory but also crossed the compromised BBB, entering the brain parenchyma. This is significant as it suggests that microclots may serve as an entry point for nanoparticles such as LNDs, potentially allowing the delivery of therapeutic agents directly to affected brain tissues. We confirmed this using MNPs-based microclots to open the BBB. Additionally, our data explain the cellular and subcellular mechanisms responsible for the biodistribution of lipid nanoparticles in the ischemic brain published earlier,^15−17^ define the exact site of passive particle retention, and suggest LNDs as potential drug carriers for the delivery of therapeutics to the brain under conditions where microthrombi are present, i.e., ischemic stroke or traumatic brain injury.^12^

To investigate the dynamic and ultrastructural insights into the biodistribution of systemically applied NPs in the brain after transient stroke, we had to develop and improve several pioneering technologies. After observing the retention of LNDs inside microclots in the ischemic brain by confocal microscopy, we used in vivo deep brain 2-photon imaging to understand the dynamics and the mechanism of this process. For this purpose, we developed a new MNPs-based stroke model, Nanostroke, which allowed us to image exactly the same cerebral vessels before and after reperfusion. The Nanostroke model is based on the recently published SIMPLE stroke model performed in pups.^13^ We adapted the SIMPLE model to adult animals by implanting a chronic cranial window, thus allowing us to visualize cerebral vessels in the ischemic brain longitudinally with high resolution. Combining the Nanostroke model with highly fluorescent, superbright FedEcs-LNDs and intravital 2-PM, we were able to demonstrate in real-time that following reperfusion systemically injected LNDs accumulate inside microthrombi and extravasate there, a so far missed phenomenon due to the use of low-fluorescence NPs. Further, the volume CLEM method we developed allowed us to match real-time 2-PM observations with ultrastructural data at a high spatial resolution. This methodology enabled us to visualize LND accumulation within microthrombi and correlate it with 3D structural features such as fibrin nets and erythrocyte morphology. Moreover, this technique enabled us to combine a dynamic component (timing of clot appearance) with ultrastructural information on BEC, thereby demonstrating that microclots compromise the function of the BBB by an increase in the formation of caveolae.

Accumulation of LNDs inside microclots after cerebral ischemia may have a large medical potential. The standard treatment for ischemic stroke is recanalizing large cerebral arteries either by dissolving or removing the occluding thrombus by systemic administration of recombinant tissue plasminogen activator or by mechanical thrombectomy, respectively.^18^ However, in many cases, recanalization of large cerebral vessels does not result in reperfusion of the cerebral microcirculation and restoration of cerebral blood flow and may thus allow additional tissue damage to occur.^19^ In previous attempts to dissolve microthrombi after stroke researchers attached the main thrombus-attaching protein (Cys-Arg-Glu-Lys-Ala) CREKA on the surface of nanocarriers,^20−22^ however, due to the lack of appropriate imaging techniques, it remained elusive whether these particles delivered their cargo into microclots. In the current study, we did not use any active targeting strategy; still, our LNDs ended up in large numbers within microclots. This passive biodistribution of LNDs in poststroke microclots is of fundamental medical importance and may pave the way for future approaches to target microthrombi with respective active compounds.

It still remains unclear how such high numbers of NPs get trapped in microthrombi. One possible explanation could be that NPs get trapped in forming microthrombi, i.e. while the clot forms; however, the large number of NPs located between all components of the clot does not seem to support such a scenario. The more likely explanation is that it takes some time until clots form and fully occlude a cerebral microvessel, as suggested by our previous findings and by others.^23−25^ During this period, which may last for minutes or even hours, vessels are not patent for cells but for plasma, and since NPs travel with plasma, they enter the clot and get trapped between accumulated fibrin fibers and stalled red and white blood cells. This “filtering” mechanism fully explains the large number of LNDs we found in microclots and the specificity with which LNDs accumulate in these structures. This concept is well in line with previous studies which showed neuroprotective effects with nanoscale compounds after ischemic stroke, but did not elaborate on the mechanisms of particle distribution and cargo delivery.^26,27^ The accumulation and subsequent extravasation of nanocarriers at the microclot site is particularly advantageous, as it halts their motion in the bloodstream, increasing the likelihood of interaction with BEC caveolae and enhancing the concentration of nanoparticles that cross the BBB, thereby maximizing their therapeutic potential in the brain parenchyma. Moreover, our findings demonstrated the critical role of the microclot microenvironment in driving the decomposition of LNDs and associated FRET loss. The degradation of LNDs in the bloodstream, likely caused by shear stress as well as interactions with serum albumin^7^ and lipoproteins,^28^ is a relatively slow process.^7^ In contrast, the entrapment of particles within microclots ceases their motion, subjecting them to prolonged exposure to specific localized conditions. Biochemical factors, such as elevated enzymatic activity (e.g., phospholipases and proteases)^29,30^ and increased oxidative stress, along with physical factors, including slightly acidic pH and mechanical compression from clot contraction, collectively could destabilize the particles and induce cargo release. However, future studies should focus on quantifying these parameters to better understand their contributions to particle decomposition and to guide the design of nanoscale lipidic systems.

In the current study, we chose LNDs as nanocarriers because they are regarded to be nontoxic and biodegradable and they have a relatively long half-life of up to 8h in mammalian blood. To monitor cargo release, we equipped LNDs with a FRET-based cargo release detection system, FedEcs. FRET systems have been widely used in nanoscience for more than a decade,^31^ however, the novelty of our current approach was to use a FRET pair compatible specifically with 2-photon excitation for high-resolution intravital imaging of cargo release. This approach resulted in LNDs with such a high FRET signal that LNDs could be visualized by 2-PM at the subcellular level in mouse blood and brain in vivo. In comparison to recent, more traditional approaches to image drug delivery to the brain using fluorescent NPs,^15,32,33^ FedEcs has the large advantage of being able to visualize the actual moment when LNDs decompose and release their cargo, even in tiny amounts of crossed BBB LNDs. Thereby, FedEcs can clearly distinguish between artifactual cargo delivery to the brain—where dyes leak from circulating nanocarriers and cross the BBB—and factual cargo delivery, where the carrier itself crosses the BBB and releases its cargo thereafter. Thus, FedEcs will allow researchers to monitor the release of cargo from individual NPs with high spatial and temporal resolution in all organs accessible to in vivo microscopy avoiding all ambiguities caused by previous methods.

Regarding the limitations of the current study, it should be noted that the Nanostroke model induces cerebral infarction in the superficial brain layer, allowing the tracking of FedEcs with intravital 2-PM at depths of up to 500 μm. Although the system is not suitable for deeper brain lesions or organs like the heart, its applicability extends to other brain diseases like glioblastoma or Alzheimer’s; systemic processes, like infection, LPS injection, and other 2-PM accessible tissues such as the lungs, liver, and spinal cord. Furthermore, its potential could be significantly enhanced by incorporating advanced imaging techniques, such as three-photon microscopy,^34^ and in vivo optical transparency methods^35^ to extend imaging depth and clarity.

## Conclusions

In conclusion, we successfully developed highly fluorescent lipid nanodroplets carrying a FRET-based cargo delivery system (FedEcs). FedEcs-LNDs are bright enough to be visualized individually in the blood and brain of living mice by 2-photon microscopy. Using in vivo brain imaging, we were able to quantify the stability of LNDs in blood and demonstrate the preferential accumulation of LNDs in microvascular clots, extravasation of intact LNDs across the blood-brain barrier, and cargo delivery to the brain parenchyma following cerebral ischemia. Therefore, FedEcs is a reliable and potent dynamic indicator of cargo delivery and release and provides a future testing ground for the efficacy and delivery of a wide range of targeted nanoscale therapy in the future.

## Material and Methods

### Formulation of LNDs

Dye-loaded nanoemulsions were obtained by spontaneous nanoemulsification. Briefly, the dyes R18-TPB (prepared as described before)^36^ were dissolved in LabrafacWL at 2% by weight. In the case of FRET-LNDs, F888 (FRET donor, prepared as described before^10^) and DiI-TPB (FRET acceptor, prepared as described before^11^) Then, 60 mg of the oil with the dye were mixed with 40 mg Cremophor ELP (also called Kolliphor ELP) and homogenized under magnetic stirring at 40 °C for 10 min. The LNDs were obtained by the addition of ultrapure (Milli-Q) water (230 mg) under rapid magnetic stirring. Size distributions were determined by dynamic light scattering using a Malvern Zetasizer ZSP.

### Animals

Male 6–8 weeks old, 18–22 g, C57Bl6/J mice from Charles River Laboratories (Sulzfeld, Bavaria, Germany) were used. Mice were group-housed under pathogen-free conditions and bred in the animal housing facility of the Institute of Stroke and Dementia Research (Muenchen, Germany), with food and water provided ad libitum (21 ± 1 °C, at 12/12 h light/dark cycle). Animal husbandry, health screens, and hygiene management checks were performed in accordance with the Federation of European Laboratory Animal Science Associations (FELASA) guidelines and recommendations.^37^ All experiments were carried out in compliance with the National Guidelines for Animal Protection of Germany with the approval of the regional Animal care committee of the Government of Upper Bavaria and were overseen by a veterinarian. The data were collected in accordance with the ARRIVE guidelines.^38^

### Stroke fMCAo

Ischemic stroke animal model via filament middle cerebral artery occlusion (fMCAo) was performed as previously described.^39^ Briefly, buprenorphine (0.1 mg/kg) injected animals, 30 min later, were anesthetized with 1.8–2% isoflurane in 50% O_2_ in the air by face mask under the control of rectal temperature (37 ± 0.1 °C) with a feedback-controlled heating pad. Regional cerebral blood flow (rCBF) over the territory of the middle cerebral artery (MCA) was monitored via a glued-onto parietal skull probe using laser Doppler fluxmetry (Perimed, Stockholm, Sweden). A silicone-coated monofilament (Doccol Corporation, Sharon, MA, USA) was introduced into the left common carotid artery and advanced toward the Circle of Willis until a drop of rCBF more than 80% of baseline indicated occlusion of the MCA. Thereafter, animals were allowed to wake up. After 60 min, animals were reanesthetized, and the filament was removed to allow reperfusion. Immediately, a femoral artery catheter was inserted and 30 min postreperfusion LNDs or MNPs were injected in volumes 4 or 6 μL/g per animal, respectively.

### Ex Vivo Imaging: Confocal (fMCAo, Nanostroke)

Animals were injected via a femoral catheter with 50 μL of DyLight 649 Labeled *Lycopersicon esculentum* (Tomato) Lectin (Vector Laboratories, Burlingame, CA, US) and 5 min later were transcardially perfused with 4% PFA in deep anesthesia. Free-floating coronal 80 μm sections were prepared as previously described.^12^ The sections were blocked and simultaneously stained with the primary antibody in buffer (1% bovine serum albumin, 0.1% gelatin from cold water fish skin, 0.5% Triton X-100 in 0.01 M PBS, pH 7.2–7.4) for 72h at 4 °C. The following primary antibodies were used: iba-1 (rabbit, Wako, #019–19741, 1:200), NeuN (guinea pig, Synaptic Systems, #266 004, 1:100), GFAP-Cy3 (mouse, Sigma-Aldrich, #2905, 1:200), Cav-1 (Cell signaling (Danvers, MA, USA), #3238, 1:100). After incubation sections were washed in PBS and incubated with the following secondary antibodies: antirabbit coupled to Alexa-fluor 488 (goat antirabbit, Thermo Fisher Scientific, 1:100), antirabbit coupled to Alexa-fluor 594 (goat antirabbit, Thermo Fisher Scientific, #A-11012, 1:200), antiguinea pig coupled to Alexa-fluor 647 (goat antiguinea pig, Thermo Fisher Scientific, #A-21450, 1:200) in secondary antibody buffer (0.05% Tween 20 in 0.01 M PBS, pH 7.2–7.4). Nuclei were stained with 4′,6-Diamidin-2-phenylindol (DAPI, Invitrogen, #D1306) 1:10,000 or DRAQ5 (ThermoFisher, #65-0880-92) 1:1000 in 0.01 M PBS. Imaging was performed using confocal microscopy (ZEISS LSM 900, Carl Zeiss Microscopy GmbH, Jena Germany). For NeuN and iba-1 quantification, a 40× magnification was used (objective: EC Plan-Neofluar 40*x*/1.30 Oil DIC M27) with an image matrix of 512 × 512 pixels, a pixel scaling of 0.4 × 0.4 μm, and a depth of 8 bit. Three different ROIs in the lesion from superficial to deeper levels were chosen and collected in 15 μm z-stacks with a slice distance of 1 μm. Homotypical areas were imaged on the contralateral hemisphere and used as controls. MAP2 intensity was then measured and normalized to contralateral.

#### Analysis of Microglia Coverage

Microglia coverage was manually assessed in a maximum intensity projection of iba-1 stained sections. ROIs were chosen in the center of the lesion at 250 μm depth from the cortex surface at −1 mm from the bregma. Homotypical ROIs were chosen on the contralesional hemisphere. Three ROIs per section per hemisphere were chosen. For microglial coverage, manual cell counting was used. To assess microglia morphology, Sholl and fractal analysis were performed to indicate ramification, cell range, and circularity using a modified protocol from Young and Morrison.^40^ Z-stack images were converted to a maximum intensity projection, and microglia cells were individually cut out using the polygon selection tool in ImageJ. Only cells that were entirely contained within the z-stack were selected. Images were then thresholded and binarized, as well as resized to 600 × 600 pixels, keeping the original scale. Any speckles and debris around the cell were removed by using the paintbrush tool. Sholl analysis was performed using ImageJ.^41^ Centered on the soma, concentric circles with an increasing radius of 1 μm were drawn, with the number of intersections measured at each radius.

After converting binary images to outlines, fractal analysis was performed using the FracLac plugin for ImageJ.^42^ As described previously,^40^ the total number of pixels present in the cell image of either the filled or outlined binary image was calculated and later transformed to μm^2^ (pixel area = 0.208 μm^2^). Cell circularity was calculated as


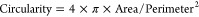


#### Statistical Analysis

All data are given as mean ± standard deviation (SD) if not indicated otherwise. For comparison between groups, the Student *t-*test was used for normally distributed data and the Mann–Whitney Rank Sum test for non-normally distributed data according to the result of the Kolmogorov–Smirnov normality test. Measurements over time were tested between groups using One-way or Two-way ANOVA with Repeated Measurements, followed by Tukey’s multiple comparisons test for normal and Sidak’s multiple comparisons test for non-normally distributed data as a post hoc test. Calculations were performed with Sigmaplot version 14.0 (Systat Software GmbH, Erkrath, Germany) and GraphPad Prism version 8.4.3 (GraphPad Software, San Diego, California, USA).

### Cranial Window Preparation

Before use, surgical tools were sterilized in a glass-bead sterilizer (FST, Heidelberg, Germany). Mice were anesthetized by an intraperitoneal injection of medetomidine (0.5 mg/kg), fentanyl (0.05 mg/kg), and midazolam (5 mg/kg) (MMF) (140/10 mg/kg body weight, WDT, Bayer, Leverkusen, Germany). Subsequently, mice were placed and head-fixed in a stereotactic frame (David Kopf Instruments, Tujunga, CA, USA). Throughout the experiment, body temperature was monitored and maintained by a rectal probe attached to a feedback-controlled heating pad (Harvard Apparatus, Holliston, MA, USA). Eyes were protected from drying by applying eye ointment (Bepanthen, Bayer, Leverkusen, Germany). The scalp was washed with swabs soaked with 70% ethanol. A flap of skin covering the cranium was excised using small scissors. The periosteum was scraped away with a 15-size scalpel (Swann Morton, Owlerton Green Sheffield, UK). The prospective craniotomy location above the primary somatosensory cortex (AP: −0.9 mm and ML: + 3.0 mm relative to bregma) was marked with a biopsy punch (diameter 3 mm, Integra LifeSciences, Princeton, New Jersey, USA). The exposed skull around the area of interest was covered with a thin layer of dental acrylic (iBond Self Etch, Hereaus Kulzer, Hanau, Germany) and hardened with an LED polymerization lamp (Demi Plus, Kerr, Orange, CA, USA). A dental drill (Schick Technikmaster C1, Pluradent Frankfurt am Main, Germany) was used to thin the skull around the marked area. After applying a drop of sterile phosphate buffered saline (DPBS, Gibco, Life Technologies, Carlsbad, CA, USA) on the craniotomy the detached circular bone flap was removed using forceps (S&T Vessel Dilating Forceps - Angled 45°, FST, Heidelberg, Germany). Subsequently, SuperGrip forceps were used to remove carefully the dura mater (S&T Forceps – SuperGrip Tips, FST, Heidelberg, Germany), and the brain was rinsed with saline. A circular coverslip (3 mm diameter, thickness #0, VWR International, Radnor, PA, USA) was placed onto the craniotomy and glued to the skull with histoacrylic adhesive (Aesculap AG, Tuttlingen, Germany), applied via dental microbrushes (0.5 mm tip diameter, Microbrush International, Grafton, WI, USA). The exposed skull was covered with dental acrylic (Tetric Evoflow A1 Fill, Ivoclar Vivadent, Schaan, Liechtenstein) and a custom-made head-post was attached parallel to the window for head-fixing mice in subsequent imaging sessions. After surgery, the anesthesia was antagonized with a combination of atipamezol (2.5 mg/kg), flumazenil (0.5 mg/kg), and naloxon (1.2 mg/kg) i.p. Finally, mice received a subcutaneous dose of the analgesic carprophen (7.5 mg/kg body weight, Rimadyl, Pfizer, New York, NY, USA) and were allowed to recover from surgery in a heating chamber.

### Intravital 2-Photon Imaging

2-Photon images were scanned as previously described.^43^ Briefly, the anesthetized mouse was placed under an upright Zeiss LSM710 microscope equipped with a fs-laser (Mai Tai DeepSee, Spectra-Physics, Stahnsdorf, Germany), a 20x water immersion objective (W Plan-Apochromat 20×/1.0 NA, Zeiss) and a motorized stage. Ti:Sa laser (Chameleon Vision II) from 917 Coherent (Glasgow, Scotland) with an excitation wavelength of 830 nm and power of 5–22% was used for detection. GAASP detector with an SP < 485 nm filter with master gain 600 for the donor channel and LP > 570 nm for the LNDs acceptor or FRET channel with master gain 600 were used. Images were taken as z-stacks (150 μm, 1024 × 1024, 8 bit, objective: W Plan-Apochromat 20×/1.0 DIC D = 0.17 M27 75 mm).

### FRET In Vivo Ratiometric Imaging

There were three types of LNDs that were used in the FRET in vivo experiment: loaded with donor (F888), loaded with acceptor (Cy3), and with both donor and acceptor (FRET). All solutions were injected in volume 7.5 μL/kg body weight into the mice with implanted cranial windows. Mice under MMF anesthesia were placed on the 2-PM stage. The LNDs were excited at 830 nm (and 740 nm for LNDs loaded with acceptor) and the emission was collected with SP < 485 nm (donor channel) and LP > 570 (acceptor/FRET channel) filters by nondescanned detectors (photomultiplier tube GaAsP, Zeiss). Three-dimensional z-stacks of 150 μm depth with 1 μm axial resolution and 1024 × 1024 pixels per image frame (0.6 μm/pixel) were acquired in multiphoton mode of the microscope over a time course of 120 min. The intensity was calculated at each channel as the total intensity of the maximum intensity projection image using Fiji software. The FRET ratio was calculated using the formula: FRETratio = FRET intensity/Donor intensity. Ratiometric analysis of LNDs integrity was performed in blood circulation 30 vs 80 nm. Statistical analysis was performed using two-way ANOVA with Tukey correction for multiple comparisons (*n* = 3 for each group) with GraphPad Prism (GraphPad Software, San Diego, CA, USA).

### Nanostroke, a Transient Mini-Stroke Mouse Model

Stroke induction started 1 day after cranial window implantation. Throughout the experimental procedure, mice were anesthetized with an intraperitoneal injection of medetomidine (0.5 mg/kg), fentanyl (0.05 mg/kg), and midazolam (5 mg/kg) (MMF) (140/10 mg/kg) and placed on a heating pad to keep body temperature at 37 °C (Harvard Apparatus, Holliston, MA, USA). The head-holder of the mouse was fixed to a custom-made headpost, and a neodymium–iron-boron (NdFeB) magnet was placed on the cranial window overlying the distal branches of the middle cerebral artery (MCA; Figure 2D, i, ii). NdFeB magnets were custom-made (Zhenli Co. Ltd., Jiaozuo, Henan, China) with the following specifications: Cylindrical NdFeB magnets, grade N52, 1 mm long, 1.5 mm in diameter, magnetized along the cylindrical axis. Before injection, all solutions with nanoparticles were mixed thoroughly using a vortex shaker (VTX-3000L, LMS). Magnetic nanoparticles, Nanomag-D (Micromod, Rostok, Germany), diameter 180 nm, PEG 2000 coated, were injected slowly at a volume of 6 μL/g body weight via a femoral artery catheter. Since iron oxide is magnetite and each particle acts as an individual magnetic domain,^44^ we expected to block the vessels via interaction between a magnet and blood-circulated MNPs. The safety, biocompatibility, and physical parameters of these MNPs were described previously.^13^ Subsequently, injected MNPs (Figure 2D, ii) into the bloodstream were trapped in the magnetic field applied to the cerebral cortex, thereby occluding the underlying vessels as evidenced by the black color filling the vessel lumen (Figure 2D, iii). Reperfusion was then induced by removing the magnet. Ten minutes thereafter, the blood flow in the distal branches of the middle cerebral artery (black arrow) as well as in most pial veins was restored (Figure 2D, iv). Vessel occlusion was monitored and recorded under a stereo microscope M80 (Leica, Wetzlar, Germany), equipped with a video camera DFC 290 HP (Leica). After 1 h the magnet was removed to induce reperfusion and a suspension of ultrabright FRET LNDs (7.5 μL/kg body weight) was injected. Systemic injection of fluorescent LNDs was performed through the same catheter used for MNPs. The platform used for performing the nanostroke featured a custom heating pad and head holder, which were compatible with the 2-PM stage. This allowed for easy relocation of the mouse to the microscope, enabling immediate intravital imaging of the reperfused area. Subsequently, mice were transferred under an LSM 7 MP microscope (Zeiss, Oberkochen, Germany). LNDs were excited with an 830 nm wavelength, laser power 5–11%, and filters, as was described above, were SP < 485 nm and LP > 570 nm for donor and FRET respectively. Three-dimensional z-stacks of 150 μm depth with 1 μm axial resolution and 1024 × 1024 pixels per image frame (0.6 μm/pixel) were acquired in multiphoton mode of the microscope every 20 min over a time course of 60 min. Throughout the imaging session, laser power was kept below 50 mW to avoid phototoxicity. Sham-operated animals received MNPs 20 min before imaging without magnet application. The microthrombi were identified visually as occluded vessels (lower-to-dark fluorescent intensity) or accumulated LNDs (higher fluorescent intensity).

### Correlative Light and ATUM Volume Scanning Electron Microscopy (CLEM)

In order to provide the exact relocation of the region of interest (ROI) from 2-PM to EM, the custom-made head post for head fixation was used (Figure S05, A, white arrow), as described.^45^ This enabled the relocation of the imaging orientation from 2-PM to EM. Immediately after two-photon imaging, mice were transcardially perfused with a mixture of 4% formaldehyde and 2.5% glutaraldehyde (Electron Microscopy Sciences, EMS) in 0.1 M sodium cacodylate buffer, pH 7.4. After 1 h perfusion, mice were decapitated and the right parietal bone with the ipsilateral window was removed. Subsequently, the mouse head was attached to the stage of a vibratome (VT1000S, Leica Biosystems) via the head post to cut the brain tissue with a thickness of 80 μm parallel to the imaging plane (Figure S05A). This way, a single brain slice containing all cortical layers and the complete somatosensory cortex was obtained. The cortical brain slice was incubated in the same fixative overnight at 4 °C, washed with PBS the next day, cut at 50 μm thickness on a vibratome, and subsequently stored in 0.1 M sodium cacodylate buffer at 4 °C until the start of the postembedding. Coarse lateral relocation of the cortical ROI was guided by magnetic nanoparticles inspected under a standard binocular (Kern) and dissected at roughly 2 × 2 mm. A standard rOTO en bloc staining protocol^46^ was applied including postfixation in 2% osmium tetroxide (EMS), 1.5% potassium ferricyanide (Sigma) in 0.1 M sodium cacodylate (Science Services) buffer (pH 7.4). Staining was enhanced by reaction with 1% thiocarbohydrazide (Sigma) for 45 min at 40 °C. The tissue was washed and incubated in 2% aqueous osmium tetroxide, washed, and further contrasted by overnight incubation in 1% aqueous uranyl acetate at 4 °C and for 2h at 50 °C. Samples were dehydrated in an ascending ethanol series and infiltrated with Epon-Araldite resin (LX112, LADD).

In order to preserve the same imaging orientation for EM from the 2-photon experiment, the block was trimmed parallel to the cortical surface. The advantage of MNPs is to be seen as label-free in light, fluorescent, and electron microscopies.^44^ Thereby, the correlation strategy relied on electron-dense magnetic particles and further anatomical landmarks like vascular patterns (Figure S05 and B) and regions of MNP leftovers in the reperfused area, far outside of the ROI. The area of interest was exposed by trimming using TRIM90 (Diatome) on the ATUMtome (Powertome, RMC). In total, 1500 serial sections were collected starting from the pial surface with a 35° ultramaxi diamond knife (Diatome) at a nominal cutting thickness and resulting axial resolution of 100 nm and collected on freshly plasma-treated (custom-built, based on Pelco easiGlow, adopted from M. Terasaki, U. Connecticut, CT), carbon nanotube (CNT) tape (Science Services). CNT tape stripes were assembled onto adhesive carbon tape (Science Services) attached to 4 in. silicon wafers (Siegert Wafer) and grounded by adhesive carbon tape strips (Science Services). EM micrographs were acquired on a Crossbeam Gemini 340 SEM (Zeiss) with a four-quadrant backscatter detector at 8 kV. In ATLAS5 Array Tomography (Fibics), wafer overview images were generated (2000 nm/pixel). In order to relocate the region imaged by light microscopy, we acquired a data set covering 10 wafers (∼200 sections, covering 20 μm depth of tissue each) at a resolution of 0.2 × 0.2 × 4 μm. This coarse EM map was aligned (TrakEM2)^42^ and different cell types were segmented (VAST).^47^ Building on this cellular map, regions of interest surrounded but devoid of magnetic particle accumulations were selected for high-resolution imaging at 20 × 20 × 100 nm voxel size. High-resolution stacks were aligned, segmented, and rendered (Blender).^48^ Accordingly, the first step was to identify the 2-PM field-of-view on a macro photograph of the cranial window (Figure S05 and C, white arrows). This ROI was identified in low-resolution EM using MNPs as fiducials and vascular patterns as endogenous landmarks (Figure S05, C, black arrows). Registration of the 2-PM and the low-resolution SEM image volumes allowed us to target time-lapse features of interest like microthrombi formation or extravasation events (Figure S05, D, blue arrows) and acquire high-resolution volume SEM data thereof.

## Supporting Information Available

Comparison of imaging of the same region of interest by electron and two-photon microscopies (MP4)
3D models of microthrombi without extravasation (MP4)
3D models of microthrombi with extravasation (MP4)
Characteristics of lipid nanodroplets (S1), ex vivo poststroke biodistribution of lipid nanodroplets (S2), characterization of nanostroke model (S3), intravital imaging of Nanostroke vs sham (S4), correlative-light electron microscopy strategy (S5), Caveolin-1 expression in occluded vessels (S6), mechanical vessel occlusion causes extravasation (S7), and comparison of lipid nanodroplets integrity 30 nm vs 80 nm (S8) (PDF)

## Notes

The following is a preprint version of our work: Igor Khalin, Nagappanpillai Adarsh, Martina Schifferer, Antonia Wehn, Valeria J. Boide-Trujillo, Uta Mamrak, Joshua Shrouder, Thomas Misgeld, Severin Filser, Andrey Klymchenko, Nikolaus Plesnila. Nanocarrier Drug Release and Blood-Brain Barrier Penetration at Post-Stroke Microthrombi Monitored by Real-Time Förster Resonance Energy Transfer-Based Detection System (FedEcs). November 24, 2024. bioRxiv 2024.02.28.582471; doi: 10.1101/2024.02.28.582471.
